# Differential Survival Gains Across Histologic Subtypes of Metastatic Renal Cell Carcinoma Across Therapeutic Eras: A SEER 2006–2022 Analysis

**DOI:** 10.51894/001c.164030

**Published:** 2026-07-31

**Authors:** Sai Tharun Reddy Gopannagari, Divya Ganisetti, Kesava Manikanta Achuta, Sushmita Mantravadi, Towfiqul Chowdhury, Sujata Kambhatla, Chadi Y. Saad

**Affiliations:** 1 Garden City Hospital

## 20

### Background

Immune checkpoint inhibitors have reshaped the therapeutic landscape of metastatic renal cell carcinoma (RCC). Although randomized trials have demonstrated survival benefits with immunotherapy-based regimens, the extent to which these advances translated into population-level survival improvements across histologic subtypes remains unclear.

### Objectives

To evaluate population-level survival trends in patients with metastatic RCC across therapeutic eras and to determine whether survival improvements in the immunotherapy era vary according to histologic subtype.

### Methods

We performed a retrospective cohort study using the Surveillance, Epidemiology, and End Results (SEER) database (2006–2022). Adults with distant-stage (metastatic at diagnosis) RCC were categorized into a targeted therapy era (2006–2015) and an immunotherapy era (2016–2022). Overall survival (OS) and cancer-specific survival (CSS) were evaluated using Kaplan–Meier methods, restricted mean survival time (RMST) analysis (τ = 36 months), and multivariable Cox proportional hazards models adjusting for age, sex, race/ethnicity, histology, surgery, radiation, and chemotherapy. The interaction between treatment era and histologic subtype was assessed. Sensitivity analyses were restricted to clear cell RCC.

### Results

Among 7,356 patients (5,814 targeted era; 1,542 immunotherapy era), median OS improved from 13 months (95% CI 13–14) to 17 months (95% CI 15–19), and median CSS improved from 14 months (95% CI 14–15) to 19 months (95% CI 17–21) (both log-rank *p* < 0.001). In multivariable analysis, diagnosis during the immunotherapy era was associated with improved OS (HR 0.80, 95% CI 0.76–0.86) and CSS (HR 0.77, 95% CI 0.73–0.83; both *p* < 0.001). At 36 months, RMST demonstrated an absolute OS gain of 1.82 months (95% CI 1.03–2.61) and CSS gain of 1.99 months (95% CI 1.18–2.79; both *p* < 0.001). Interaction analysis revealed reduced survival benefit in papillary and chromophobe RCC. Findings remained consistent in sensitivity analyses restricted to clear cell RCC (HR 0.78, 95% CI 0.73–0.83).

### Conclusions

Diagnosis during the immunotherapy era was associated with significant population-level survival improvement in metastatic RCC, with gains driven predominantly by clear cell histology. Our results indicate that survival gains have been uneven across histologic subtypes, reinforcing the need for more effective, subtype-specific approaches in non–clear cell RCC.

